# Alignment rod and gap measurement methods achieve comparable alignment correction in opening wedge high tibial osteotomy for varus osteoarthritic knees

**DOI:** 10.1002/jeo2.70038

**Published:** 2024-10-16

**Authors:** Shintaro Onishi, Youngji Kim, Hiroshi Nakayama, Christophe Jacquet, Ahmed Mabrouk, Matthieu Ollivier

**Affiliations:** ^1^ APHM, CNRS, ISM, Department of Orthopaedics and Traumatology, Sainte‐ Marguerite Hospital, Institute for Locomotion Aix Marseille University Marseille France; ^2^ Department of Orthopaedic Surgery Hyogo Medical University Nishinomiya Japan; ^3^ Department of Orthopaedics Juntendo University Faculty of Medicine Tokyo Japan; ^4^ Department of Trauma and Orthopaedics Leeds Teaching Hospitals Leeds UK

**Keywords:** accuracy of correction angle, alignment rod, gap measurement, opening wedge high tibial osteotomy

## Abstract

**Purpose:**

To compare clinical and radiological outcomes of medial opening wedge high tibial osteotomy (MOWHTO) using two different alignment methods: the alignment rod (AR) versus the gap measurement (GM) method. The primary outcome was to report the surgical accuracy of coronal plane corrections in each method.

**Methods:**

Patients who underwent MOWHTO with either AR or GM method between 2014 and 2022 at a single institution, with a minimum of 2 years of follow‐up, were included. The opening gap was gradually spread with an AR under fluoroscopic control in the AR group, whereas the osteotomy site was opened to the value of the measured gap distance in addition to the thickness of the bone saw in the GM group. Radiological assessment of geometric characteristics included hip–knee–ankle angle (HKA), medial proximal tibial angle (MPTA), mechanical lateral distal femoral angle and joint line convergence angle. Surgical accuracy, which is the deviation (Δ) between the intended and achieved correction, was compared between both methods. Clinical outcomes were assessed using the Knee Injury and Osteoarthritis Outcome Scores.

**Results:**

A total of 110 patients (*n* = 110 knees) with a mean age of 54.1 ± 8.4 years were included in the study. Radiological parameters were significantly improved as reflected by HKA correction from 171.6° ± 2.0° to 181.1° ± 2.6° in the AR group and from 171.0° ± 2.3° to 181.1° ± 2.8° in the GM group at 2 years (Intergroup n.s). There was no significant intergroup difference for all radiological parameters and clinical outcomes. There was no intergroup difference in the surgical accuracy as evaluated by Δvalues and absolute Δvalues of both HKA and MPTA (n.s).

**Conclusions:**

Comparable correction accuracy was achieved in MOWHTO using either the AR or GM method. The GM method is simple and reliable in achieving the intended correction in MOWHTO.

**Level of Evidence:**

Ⅲ retrospective comparative study.

**Abbreviations**
ADL: activities of daily living
AR: alignment rod
BMI: body mass index
CAS: computer‐assisted navigation systems
GM: gap measurement
HKA: hip–knee–ankle angle
HTO: high tibial osteotomy
JLCA: joint line convergence angle
KOOS: knee injury and osteoarthritis outcome score
mLDFA: mechanical lateral distal femoral angle
mMPTA: mechanical medial proximal tibial angle
MOWHTO: medial opening wedge high tibial osteotomy
MPTA: medial proximal tibial angle
OA: osteoarthritis
PSI: patient‐specific instrumentation
QOL: quality of life

## INTRODUCTION

Osteotomies around the knee are an established treatment modality for active patients with unicompartmental knee osteoarthritis (OA). Medial opening wedge high tibial osteotomy (MOWHTO) is widely performed among the various high tibial osteotomy (HTO) procedures [4, 12, 21, 24]. Numerous studies have reported that optimal alignment is a critical factor in achieving good clinical outcomes after MOWHTO [4, 6, 33]. The surgical concept of osteotomies around the knee is to restore optimal bone and joint geometry, resulting in a favourable biomechanical environment at the knee joint [19, 35]. Therefore, it is mandatory to correct the deformity precisely according to the preoperative plan. However, a substantial rate of postoperative correction error after MOWHTO has been reported [3, 10]. Regarding intraoperative correction, the use of computer‐assisted navigation systems (CAS) and patient‐specific instrumentation (PSI) has been proposed and proven to be effective methods to reduce bony correction errors during surgery [5, 9, 23]. Despite superior correction accuracy and fewer related intraoperative complications, the cost‐effectiveness of the CAS and PSI remains controversial [1, 29, 37].

Using an alignment rod (AR) under fluoroscopic guidance is a conventional and widely used method in daily clinical practice to achieve adequate intraoperative correction with the advantage of not requiring any specific equipment [15]. Additionally, the accuracy of measurements and their reliability have improved with the introduction of preoperative planning using digital planning software [30, 34]. On the other hand, preoperative gap measurement (GM) using a digital system is known to be a precise and applicable modality in knee osteotomies [7, 29]. However, there is a paucity of information regarding the differences in the accuracy of coronal alignment correction between AR and GM methods for MOWHTO.

The purpose of this study was to compare the clinical and radiological outcomes after MOWHTO using either the AR or GM method. The primary outcome was to report the surgical accuracy of coronal plane correction in each method. It was hypothesized that there is no difference between the two methods.

## METHODS

### Study population and design

**Figure 1:**
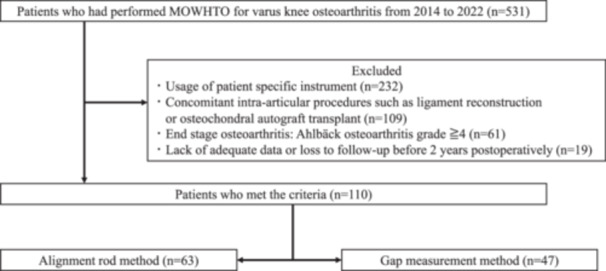
Flowchart of the patient selection process.

After institutional review board approval (IRB PADS24‐171_dgr), a retrospective analysis of a prospectively maintained database was performed. Five hundred and thirty‐one consecutive knees with varus knee OA who underwent MOWHTO at a single, tertiary referral centre from 2014 to 2022. The inclusion criteria for the present study were isolated MOWHTO, age range of 18–70 years and no prior surgery except arthroscopic meniscus repair or meniscectomy. Patients with end‐stage OA (Ahlbäck OA grade ≧4) and concomitant intra‐articular procedures such as ligament reconstruction or osteochondral autograft transplantation and cases where PSI was used were excluded. Also, cases with inadequate data or who lost to follow‐up before the second postoperative year were excluded. The patient flow chart is presented in Figure 1.

### Surgical options and procedures

Surgeries were performed by two senior surgeons in a single institution. Indication for surgery was decided based on the radiological assessment of the whole lower limb alignment and bone/joint geometries. MOWHTO was indicated for patients who primarily had an extra‐articular tibial deformity with a medial proximal tibial angle (MPTA) of less than 85° and with intra‐articular pain in the medial compartment [25]. Prior to surgery, radiological evaluation and surgical planning were conducted on a weight‐bearing long‐limb radiograph using measurement tools within the Centricity Universal Viewer Zero Footprint (General Electric Healthcare) imaging software. The weight‐bearing line was planned to pass through a point 50%–57% from the medial tibial plateau, depending on the patient's joint geometry. The correction angle was calculated for all cases using Miniaci's method [22]. However, in terms of actual correction during surgery, the intraoperative alignment using AR under fluoroscopic control was preceded in the AR group.

**Figure 2:**
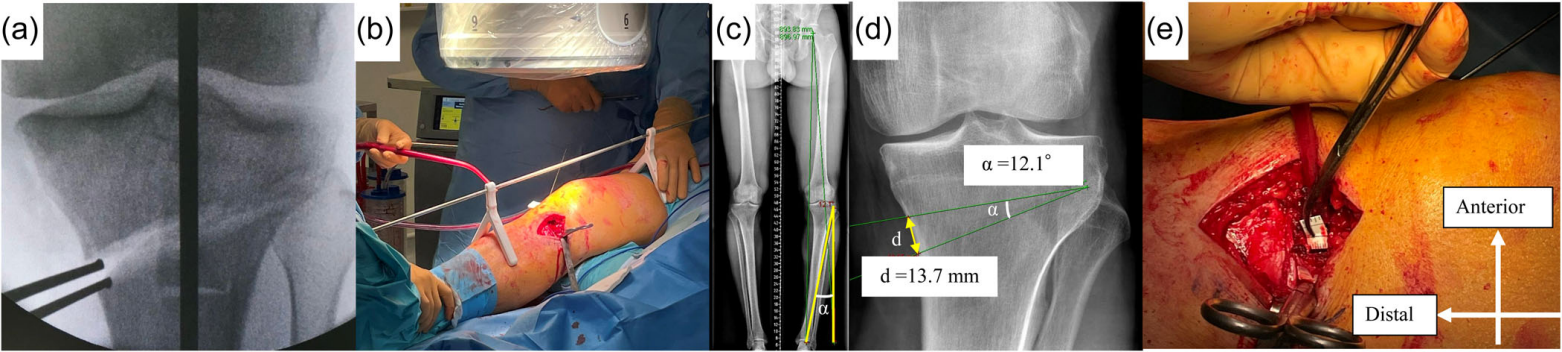
Alignment correction using both the AR method and GM method. (a, b) The AR is placed along the centre of the femoral head and ankle under fluoroscopy. The medial osteotomy site is spread until the rod is passed through an intended point on the knee joint. (c, d) The gap height is measured preoperatively using the Miniaci method, taking into account the thickness of the bone saw (1 mm). In this representative case, the actual gap is estimated to be 14.7 mm. (e) The intended value of the gap height is used for intraoperative correction without using AR. AR, alignment rod; GM, gap measurement.

Surgeries were performed under general anaesthesia or spinal anaesthesia. A pneumatic tourniquet was used during surgery. Prior to the osteotomy, arthroscopic examination and procedures for intra‐articular pathologies such as meniscal lesions were performed in most cases as needed. The MOWHTO procedure in the study population followed the biplanar osteotomy technique as described in previous studies [13, 20]. Briefly, a medial approach was made to the proximal tibia with an oblique skin incision and exposure for biplanar osteotomy was performed. Double soft tissue windows were then created for retraction of the pes anserinus posteriorly, as well as placement of the posterior tissue retractor to protect the neurovascular bundle. The biplanar osteotomy cuts were performed, ensuring that the horizontal cut was just proximal to the tibiofibular joint level and the sagittal cut was directed cranially under fluoroscopic control. For the AR method, the metal rod was placed along the centre of the femoral head and ankle under fluoroscopy. The medial osteotomy site was spread using a laminar bone spreader until the rod was passed through an intended point on the knee joint. Once optimal alignment was achieved, the metal plate was fixed while maintaining the corrected status (Figure 2a,b). Regarding the GM method, intended opening gap was measured preoperatively using Miniaci's method, all while taking into account the thickness of the bone saw. The intended value of the gap height was applied to intraoperative correction without the use of AR (Figure 2c,d,e). In all cases, the Activmotion® HTO plate (Newclip Technics) was used, and allogenic bone graft was placed to fill the osteotomy gap for all cases. The choice of AR or GM method was made based on the surgeon's preference during the study period.

### Postoperative rehabilitation

All patients in both groups were allowed immediate postoperative full range of motion and weight bearing as tolerated using crutches if there was no intraoperative lateral hinge fracture. Whereas, if a lateral hinge fracture was identified intraoperatively, non‐weight bearing or toe touch weight bearing was recommended, depending on the type of lateral hinge fracture [36]. Postoperatively, low‐molecular‐weight heparin was used for thromboprophylaxis for 30 days. Return to sports activities was recommended after the confirmation of adequate muscle strength and solid bony healing at the osteotomy site.

### Radiographic measurements and clinical outcomes

Pre‐ and postoperative radiological and clinical data at 2 years were retrospectively reviewed. The conventional knee radiographs were assessed for OA grade based on the Kellgren–Lawrence and Ahlbäck classifications. On the long leg alignment radiographs, the following alignment parameters were evaluated: the hip–knee–ankle angle (HKA), mechanical lateral distal femoral angle (mLDFA), joint line convergence angle (JLCA) and MPTA were measured preoperatively and 2 years postoperatively as parameters for analysis. As for the correction‐related parameters during preoperative planning, the intended correction angle of both HKA and MPTA and the intended opening gap distance were calculated. To evaluate the surgical accuracy after MOWHTO, the mean difference (Δ) between the intended and 2‐year follow‐up radiological parameters was calculated. To reduce the possibility that the magnitude of overcorrections could counteract that of the under‐corrections, resulting in a mean close to zero, the absolute values of Δ HKA and ΔMPTA were also assessed. Clinical outcomes were assessed using the Knee Injury and Osteoarthritis Outcome Score (KOOS) and the Tegner activity scale at 2 years after surgery.

### Statistical analysis

All statistical analyses were performed using version 18 of SPSS (SPSS Inc.). The normality of data distribution was assessed using the Shapiro–Wilk test. Statistical comparisons between groups were made using the unpaired *t*test for parametric data, the Mann–Whitney *U* test for nonparametric variables and the *χ* ^2^ test for categorical outcomes, with the significant level set at or below 0.05. All variables were statistically compared between the AR and GM groups. The accuracy of the measurements was assessed using the intraclass correlation coefficient for intra‐ and interobserver reliability; two independent observers reviewed the preoperative and postoperative radiograph sets twice in a blinded fashion with a 3‐week measurement interval: all intraclass and interclass correlation coefficients were >0.8.

A post hoc power analysis confirmed that with 42 patients in each group, it was possible to detect difference >1.5° in Δ mMPTA between the two groups with a statistical power of 80%.

## RESULTS

A total of 110 patients (*n* = 110 knees) were included in the study. The mean age in the AR group was 53.9 ± 9.2 years versus 54.4 ± 7.2 years in the GM group (n.s). Patient demographics are demonstrated in Table 1. Significant preoperative to postoperative corrections were achieved in the HKA from 171.6° ± 2.0° to 181.1° ± 2.6° in the AR group and from 171.0 ± 2.3° to 181.1° ± 2.8° in the GM group (*p* < 0.001), intergroup (n.s) (Table 2).

**Table 1:** Clinical characteristics.

	Overall (*n* = 110)	AR (*n* = 63)	GM (*n* = 47)	*p* Value
Age (years)	54.1 ± 8.4 (19–69)	53.9 ± 9.2 (19–69)	54.4 ± 7.2 (31–68)	0.74
Male/female (female, %)	92/18 (16.4%)	52/11 (17.5%)	40/7 (14.9%)	0.72
BMI (kg/m^2^)	27.8 ± 4.0 (21.4–41.2)	28.3 ± 4.4 (21.4–41.2)	27.3 ± 3.5 (21.5–35.1)	0.19
Follow‐up period (year)	4.9 ± 3.3 (2–10)	5.3 ± 3.3 (2–10)	4.2 ± 3.2 (2–10)	0.07
Preoperative Kellgren–Lawrence grade (0/1/2/3/4, %)	0/11/26/49/24 (0/10/23.6/44.5/21.8)	0/9/15/30/9 (0/14.3/23.8/47.6/14.3)	0/2/11/19/15 (0/4.3/23.4/40.4/31.9)	0.14
Preoperative Ahlbäck grade (1/2/3/4/5, %)	17/66/27/0/0 (15.5/60/24.5/0/0)	8/38/17/0/0 (12.7/60.3/27.0/0/0)	9/28/10/0/0 (19.1/59.6/21.3/0/0)	0.90

*Note*: Values are expressed as mean and standard deviations, with ranges in parentheses.

Abbreviations: AR, alignment rod; GM, gap measurement; BMI, body mass index.

**Table 2:** Comparison of pre‐ and postoperative radiological parameters between AR and GM groups.

		AR (*n* = 63)	GM (*n* = 47)	*p* Value
HKA	Preoperative	171.6 ± 2.0	171.0 ± 2.3	0.22
	Postoperative	181.1 ± 2.6	181.1 ± 2.8	0.92
mLDFA	Preoperative	89.1 ± 1.6	89.5 ± 1.8	0.18
	Postoperative	89.4 ± 1.5	89.7 ± 2.2	0.41
JLCA	Preoperative	2.9 ± 2.0	2.5 ± 1.9	0.62
	Postoperative	2.2 ± 1.8	2.4 ± 2.7	0.78
mMPTA	Preoperative	83.2 ± 2.2	83.0 ± 2.1	0.65
	Postoperative	92.2 ± 2.9	92.6 ± 3.2	0.50

*Note*: Values are expressed as mean and standard deviations.

Abbreviations: AR, alignment rod; HKA, hip–knee–ankle angle; GM, gap measurement; JLCA, joint line convergence angle; MOWHTO, medial opening wedge high tibial osteotomy; mLDFA, mechanical lateral distal femoral angle; mMPTA mechanical medial proximal tibial angle.

Similarly, there was no intergroup difference in the planned correction or the correction accuracy evaluated by the values and absolute values of ΔHKA and Δ MPTA (n.s.) (Tables 3, 4). Clinically, there was no intergroup difference in the KOOS and Tegner activity scores (n.s.) (Table 5).

**Table 3:** Comparison of the intended correction‐related radiological parameters after MOWHTO between AR and GM groups.

	Intended correction angle of the HKA (degree)	Intended correction angle of the mMPTA (degree)	Intended opening gap of the posteromedial cortex (mm)
AR (*n* = 63)	9.3 ± 3.1	9.0 ± 3.6	N.A
GM (*n* = 47)	10.0 ± 3.3	9.6 ± 3.5	11.5 ± 4.1
*p* Value	0.26	0.38	N.A

*Note*: Values are expressed as mean and standard deviations.

Abbreviations: AR, alignment rod; GM, gap measurement; HKA, hip–knee–ankle angle; MOWHTO, medial opening wedge high tibial osteotomy; mMPTA, mechanical medial proximal tibial angle.

**Table 4:** Comparison of radiological accuracy after MOWHTO between AR and GM groups.

	Δ HKA	Absolute values of the Δ HKA	Δ mMPTA	Absolute values of the Δ mMPTA
AR (*n* = 63)	1.2 ± 2.7	2.5 ± 1.5	1.7 ± 3.1	2.9 ± 2.0
GM (*n* = 47)	1.2 ± 2.8	2.6 ± 1.6	1.5 ± 3.4	2.9 ± 2.3
*p* Value	0.99	0.87	0.73	0.99

*Note*: Δ HKA and Δ mMPTA were defined as a deviation from the intended value. Values are expressed as mean and standard deviations.

Abbreviations: AR, alignment rod; GM, gap measurement; HKA, hip–knee–ankle angle; mMPTA, mechanical medial proximal tibial angle; MOWHTO, medial opening wedge high tibial osteotomy.

**Table 5:** Postoperative KOOS subscore and Tegner activity scale between AR and GM groups.

	AR (*n* = 63)	GM (*n* = 47)	*p* Value
Postoperative KOOS			
Symptoms	81.3 ± 16.4	79.5 ± 17.5	0.56
Pain	82.6 ± 13.3	83.6 ± 14.6	0.70
ADL	78.4 ± 15.6	80.6 ± 14.3	0.50
Sports/rec	77.1 ± 20.7	76.1 ± 18.6	0.65
QOL	87.1 ± 12.7	86.3 ± 12.2	0.73
Tegner activity scale	3.6 ± 1.8	3.6 ± 1.7	0.99

*Note*: Values are expressed as mean and standard deviations.

Abbreviations: AR, alignment rod; GM, gap measurement; ADL, activities of daily living; AR, alignment rod; GM, gap measurement; KOOS, Knee Injury and Osteoarthritis Outcome Score; QOL, quality of life.

## DISCUSSION

The main finding in the presented study is demonstrating no difference between the AR and GM methods in achieving significant surgical accuracy of coronal plane corrections following MOWHTO for varus osteoarthritic knees. Additionally, no significant differences were demonstrated between both methods in radiological corrections or improved clinical outcomes.

Osteotomies around the knees aim to shift the load from the affected arthritic compartment to the opposite healthy compartment [35]. A previous study showed that 1° of coronal malalignment may result in an additional 12% body weight distributed to the medial compartment [11]. Less accurate alignment with either under‐ and over‐correction has been reported as an important cause of unsatisfactory clinical outcome after HTO [8, 18, 26]. Thus, accuracy in HTO translates into better clinical outcomes.

Using a rod or a cable under fluoroscopy guidance has been well utilized as a method of evaluating the mechanical axis of the lower limb, despite being performed in a non‐weight‐bearing status [14, 15]. On the other hand, the GM technique using digital images has demonstrated higher reproducibility and reliability [7, 22, 29, 30]. Moreover, this reliability and reproducibility have been demonstrated to be comparable using either picture archiving and communication systems or commercially based digital planning software [7]. Therefore, the GM technique is readily available to all surgeons even with no additional software.

Several studies compared osteotomy correction accuracy utilizing several techniques such as GM using digital software, CAS and PSI [1, 2, 5]. However, only a few articles compared the surgical accuracy of MOWHTO using either the AR or the GM method. Yoon et al. [38] reported on the surgical accuracy of the cable method versus Miniaci's method with a relatively small number of subjects. Yoon et al. reported that Miniaci's method is more accurate than the cable method due to postural differences which result in undercorrection when using the cable method. However, in this study, there was no difference in coronal plane correction accuracy between the AR and GM methods in a larger cohort of patients. The discrepancy of the results between the two studies could be due to the material properties of the cable, used by Yoon et al. being flexible and easily bent, making the technique less reproducible and less accurate when compared with a metal rigid rod. Furthermore, the thickness of the bone saw (approximately 1 mm) was notably considered and applied to the intended GM in the presented study.

The accuracy of the GM methods is still controversial when compared with other techniques. Ribeiro et al. [28] showed that there were no differences in the accuracy of coronal alignment correction between the GM method and CAS. In addition, Schröter et al. [29] also reported the correction accuracy using the GM technique to be comparable to that of CAS. In their study, the mean surgical accuracy of coronal alignment correction was 1.7° ± 1.2° and 2.1° ± 1.4° for the GM and CAS methods, respectively. Furthermore, a previous meta‐analysis demonstrated that the use of CAS and PSI led to significantly reduced postoperative outliers when compared with the conventional technique. However, the result of this analysis did not lead to a statistically significant improvement in the accuracy of postoperative alignment [5]. In addition, a recent study showed that using the conventional GM technique was as accurate as PSI in the hands of experienced surgeons, with a mean HKA deviation of 1.3° ± 0.7° for PSI and 1.5° ± 0.9° for the conventional method [1].

Despite demonstrating high surgical accuracy in the presented series, correction errors were reported. In those cases, a failure to cope with intraarticular deformities can be a factor in eliciting correction errors, and it has been noted that excessive medial and lateral soft tissue laxity appearing in large JLCAs may be a potential source. Previous clinical studies have shown that a large JLCA is associated with postoperative mal‐correction after MOWHTO [16, 17, 31, 32]. In the presented series, the influence of preoperative JLCA on the correction accuracy could not be eliminated. However, there were no differences in pre‐ and postoperative JLCA between the two groups.

There limitations of the present study include its retrospective nature and the absence of randomization. First, potential factors influencing surgical accuracy other than bone and joint deformities such as lateral hinge fractures, divergence of osteotomy height and hinge height were not considered in the analysis. Second, long leg alignment radiographs were not assessed immediately after surgery in an attempt to exclude the influence of early postoperative pain and swelling, which may be caused by flexion contracture or insufficient weight bearing. Consequently, the chronological change in radiological parameters during the postoperative period could not be evaluated. Third, compensation for coronal alignment by the ankle and hip joints [27], which may affect the overall lower limb alignment, was not considered in the analysis. Fourth, sagittal alignment was not evaluated in this study, and surgical accuracy with respect to sagittal alignment remains unclear. In addition, surgeons may use a combination of preoperative GM and intraoperative AR in clinical practice. However, this study did not assess the surgical accuracy of these combined methods. Instead, it compared the accuracy of the AR method with that of the preoperative GM method. The findings suggest that the preoperative GM method alone can determine the necessary correction without relying on intraoperative AR. Fifth, the use of either the AR or GM method in this study was solely based on the surgeon's preference. Thus, there may be a risk of bias that the results of this study depend on the competence of the surgeon. Finally, all surgeries were performed by experienced high‐volume osteotomy surgeons. Therefore, caution should be exercised when extrapolating the results of the present study to nonexperienced or low‐volume surgeons.

Although there were limitations in the present study, this is the first study that compares radiological corrections, correction accuracy and clinical outcomes of MOWHTO using the AR versus GM methods. Further prospective studies involving a larger sample size are needed to establish optimal management strategies to avoid surgical corrective errors in osteotomies around the knees.

## CONCLUSIONS

Comparable correction accuracy was achieved in MOWHTO using either the AR or GM method. The GM method is simple and reliable in achieving the intended correction in MOWHTO.

## AUTHOR CONTRIBUTIONS

All authors have contributed to the design, content and writing of the manuscript. All authors read and approved the final manuscript.

## CONFLICTS OF INTEREST STATEMENT

M.O. has received consulting fees from Newclip Technics, Arthrex and Stryker. The remaining authors declare no conflict of interest.

## ETHICS STATEMENT

Ethical approval for this study was obtained from the Institutional Review Board in our institution. Informed consent was obtained from all individual participants included in the study.

## Data Availability

The data that support the findings of this study are available from the corresponding author upon reasonable request.
